# The diagnostic efficacy of seven autoantibodies in early detection of ground-glass nodular lung adenocarcinoma

**DOI:** 10.3389/fonc.2024.1499140

**Published:** 2024-11-26

**Authors:** Hua Guo, Wei Zhao, Chunsun Li, Zhen Wu, Ling Yu, Miaoyu Wang, Yuanhui Wei, Zirui Wang, Shangshu Liu, Yue Yin, Zhen Yang, Liangan Chen

**Affiliations:** ^1^ Medical School of Chinese People’s Liberation Army, Beijing, China; ^2^ Department of Pulmonary and Critical Care Medicine, Chinese People’s Liberation Army General Hospital, Beijing, China; ^3^ School of Medicine, Nankai University, Tianjin, China

**Keywords:** autoantibodies, ground-glass nodules, lung adenocarcinoma, diagnosis, early detection

## Abstract

**Background:**

Persistent ground-glass nodules (GGNs) carry a potential risk of malignancy, however, early diagnosis remained challenging. This study aimed to investigate the cut-off values of seven autoantibodies in patients with ground-glass nodules smaller than 3cm, and to construct machine learning models to assess the diagnostic value of these autoantibodies.

**Methods:**

In this multi-center retrospective study, we collected peripheral blood specimens from a total of 698 patients. A total of 466 patients with ground-glass nodular lung adenocarcinoma no more than 3cm were identified as a case group based on pathological reports and imaging data, and control group (n=232) of patients consisted of 90 patients with benign nodules and 142 patients with health check-ups. Seven antibodies were quantified in the serum of all participants using enzyme-linked immunosorbent assay (ELISA), and the working characteristic curves of the subjects were plotted to determine the cut-off values of the seven autoantibodies related ground-glass nodular lung adenocarcinoma early. Subsequently, the patients were randomly divided into a training and test set at a 7:3 ratio. Eight machine-learning models were constructed to compare the diagnostic performances of multiple models. The model performances were evaluated using sensitivity, specificity, and the area under the curve (AUC).

**Results:**

The serum levels of the seven autoantibodies in case group were significantly higher than those in the control group (P < 0.05). The combination of the seven autoantibodies demonstrated a significantly enhanced diagnostic efficacy in identifying ground-glass nodular lung adenocarcinoma early when compared to the diagnostic efficacy of the autoantibodies when used respectively. The combined diagnostic approach of the seven autoantibodies exhibited a sensitivity of 84.05%, specificity of 91.85%, and AUC of 0.8870, surpassing the performance of each autoantibody used individually. Furthermore, we determined that Sparrow Search Algorithm-XGBoost (SSA-XGBOOST) had the best diagnostic performance among the models (AUC=0.9265), with MAGEA1, P53, and PGP9.5 having significant feature weight proportions.

**Conclusions:**

Our research assessed the diagnostic performance of seven autoantibodies in patients with ground-glass nodules for benign-malignant distinction, and the nodules are all no more than 3cm especially. Our study set cut-off values for seven autoantibodies in identifying GGNs no more than 3cm and constructed a machine learning model for effective diagnosis. This provides a non-invasive and highly discriminative method for the evaluation of ground-glass nodules in high-risk patients.

## Introduction

In light of heightened health concerns and the growing popularity of lung cancer screening, alongside advancements in high-resolution computed tomography (CT), the detection rate of lung nodules has increased dramatically, resulting in concomitant overexposure to radiation and heightened anxiety (1, 2). Timely and accurate diagnosis of lung nodules is critically important for the early detection of lung cancer, the enhancement of overall prognosis, and the alleviation of the burden associated with medication overuse. While most incidentally detected small lung nodules are benign, persistent pulmonary ground-glass nodules (GGNs) are associated with a risk of malignancy. GGNs represent a common and distinct clinical challenge characterized by specific imaging features (3–5). These nodules are frequently indeterminate and lack the typical signs used to ascertain their nature, exhibiting a more complex and varied etiology compared to other types of lung nodules. The relatively inert nature of GGNs, their insidious progression, and the potential for improved prognosis suggest that lung cancers associated with GGNs may exhibit specific biological features (6, 7). Consequently, distinguishing between benign and malignant GGNs poses a significant challenge.

Following the development of malignant GGNs, tumor-associated antigens (TAAs) can stimulate the immune system to produce specific antibodies at an early stage (8–10). Some studies have demonstrated that autoantibodies can be identified as early as five years prior to the detection of tumors via computed tomography (CT), and they can be found in peripheral blood for an extended and consistent duration before overt clinical symptoms manifest (9, 11, 12). Traditional tumor markers, including carcinoembryonic antigen (CEA), squamous cell carcinoma antigen (SCCA), and neuron-specific enolase (NSE), have been utilized in clinical practice for a considerable duration (10, 13). However, their specificity and early diagnostic value are limited (14). Numerous studies have indicated that autoantibodies exhibit variable sensitivity and specificity across different co-diagnostic modalities. Among the more established autoantibody panels validated across multiple centers is the European EarlyCDT-Lung (15, 16) (p53, NY-ESO-1, GBU4-5, CAGE, SOX2, HuD and MAGEA4), which has entered Phase IV development. Furthermore, various autoantibody panels have also been validated in multiple populations (13, 14, 17). Nevertheless, antibody studies have been conducted in populations with lung cancer, including patients with stage IV lung cancer. The clinical cut-off values provided were not specific to GGNs associated with suspected lung cancer. Consequently, the clinical value of the seven autoantibodies for early screening of patients with early-stage ground-glass nodular lung adenocarcinoma remains inadequately investigated. Furthermore, physicians primarily rely on CT features to ascertain the benign or malignant nature of a nodule in clinical practice; thus, non-invasive and effective diagnostic marker panels are urgently needed.

The objective of our study was to determine the optimal cut-off value for seven autoantibodies (7-TAAbs: p53, PGP9.5, SOX2, GAGE7, GBU4-5, MAGEA1, and CAGE) in identifying patients with ground-glass nodular lung adenocarcinoma (≤3 cm). This objective would facilitate a more timely and accurate diagnosis for patients with stage I lung cancer. To this end, a prospective study was conducted in which the seven autoantibodies were measured in both the case and control groups. Eight machine learning models were constructed with the aim of selecting the model that demonstrated the best diagnostic performance, thereby providing an effective tool for diagnosing patients with early-stage ground-glass nodules.

## Materials and methods

### Participants

**Figure 1:**
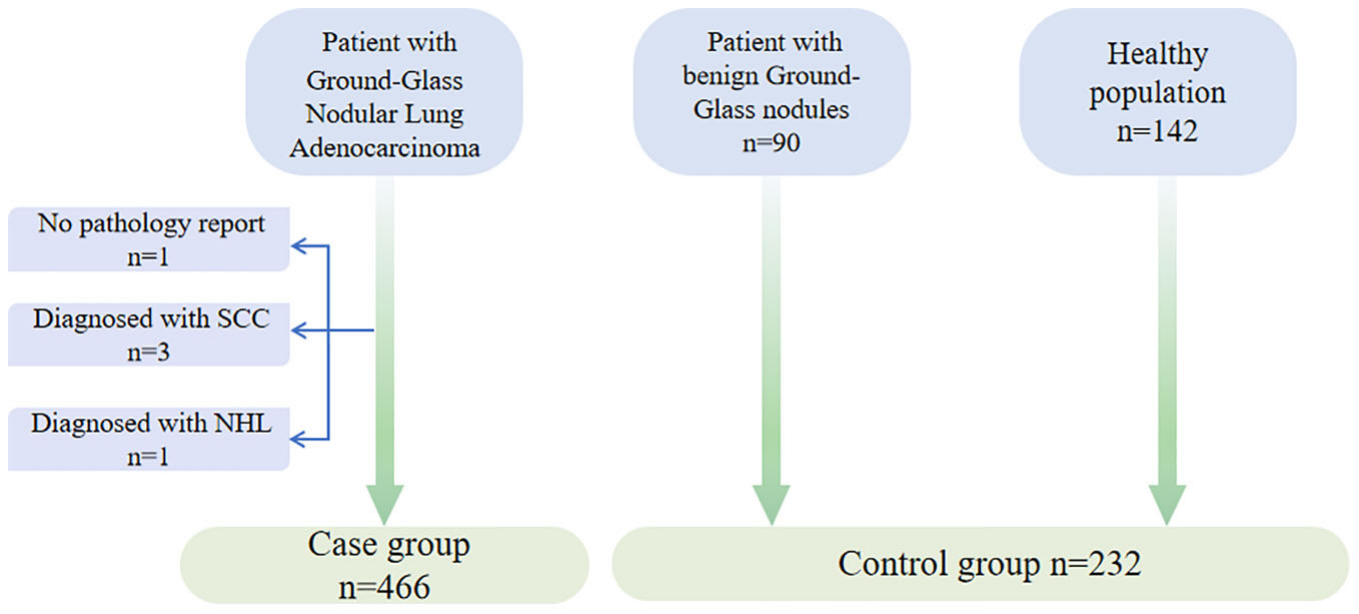
Generalization of study participant enrollment.

This study included 466 patients with ground-glass nodular lung adenocarcinoma, enrolled from August 2021 to December 2023 at multiple hospitals in China (**Figure 1**). The participants in the case group were from the First Medical Centre (n=355), the Fourth Medical Centre (n=73), and the Sixth Medical Centre (n=3) of the General Hospital of the People’s Liberation Army (PLA), as well as four hospitals in other provinces in China (n=35), including multiple hospitals in Chongqing (n=2), Qingdao (n=6), Dalian (n=10), and Qinhuangdao (n=17). Enrollment was based on the following criteria: 1) Written informed consent was signed by the patient or their legal representative. 2) Age was between 18 and 80 years. 3) Clinically detected pulmonary ground-glass nodular lesions measuring no more than 3 cm. 4) Diagnosis of ground-glass nodular lung adenocarcinoma was established via minimally invasive biopsy (respiratory endoscopic biopsy or CT-guided lung puncture biopsy) or pathology from surgical procedures. Individuals meeting any of the following exclusion criteria were excluded from the study: 1) Patients who failed to obtain a pathological diagnosis. 2) A previous history of lung cancer. 3) Patients who declined to participate in the study.

Additionally, the study included 90 patients with benign lung nodules from the First Medical Centre, all of whom met the criteria for inclusion in the following group: 1) Written informed consent was signed by the patient or their legal representative; 2) Age was between 18 and 80 years; 3) Clinical diagnosis of lung infection, benign pulmonary nodule, interstitial pneumonia, hamartoma, or other lung diseases, with imaging showing ground-glass nodules that improved significantly after treatment. Individuals meeting any of the following exclusion criteria were excluded from the study: 1) CT presentations that are not indicative of ground-glass nodules; 2) A previous history of lung cancer; 3) Patients who declined to participate in the study.

We also enrolled 142 healthy individuals from the health check-up population at the First Medical Centre of the PLA General Hospital, with the following inclusion criteria: 1) Written informed consent was signed by the patient or their legal representative; 2) Age was between 18 and 80 years; 3) The lung CT scan did not reveal any significant abnormalities, excluding benign or malignant tumors and other lung diseases; 4) Ultrasound, electrocardiogram, and other imaging tests demonstrated no discernible abnormalities.

This study was approved by the Medical Ethics Committee and institutional review board of The General Hospital of the People’s Liberation Army (Ethical number: S2020-173-01).

### Quantitation of 7-TAAbs in serum samples

Peripheral blood samples were collected and centrifuged at 3,000 rpm for 15 minutes at 4°C. Serum was collected and subsequently stored at a temperature of -80°C. A comprehensive pathological examination was conducted for all participants, encompassing demographic data and smoking history. Serum antibody concentrations were determined using enzyme-linked immunosorbent assay (ELISA) with seven autoantibody test kits manufactured by Cancer Probe Biological Technology Company. The absorbance was measured at an optical density (OD) of 450 nm using a spectrophotometer.

### Statistical analysis

The cut-off values for the seven autoantibodies were determined using receiver operating characteristic (ROC) curves, calculating the Youden index (which ranges from 0 to 1, with larger values indicating better diagnostic performance) and the area under the curve (AUC) for the respective subjects’ operating characteristic curves. All data were processed using SPSS version 26.0 statistical software. Categorical variables were expressed as counts (with percentages) and analyzed using the chi-square test or Fisher’s exact test. Continuous variables were analyzed using the t-test, while variables that do not conform to a normal distribution were tested using the Mann-Whitney U test and the Kruskal-Wallis test, with statistical significance set at P < 0.05. Scatter plots illustrating the expression of seven autoantibodies in serum were generated, and differences were calculated using GraphPad Prism version 10.1.

### Machine learning methods

**Figure 2:**
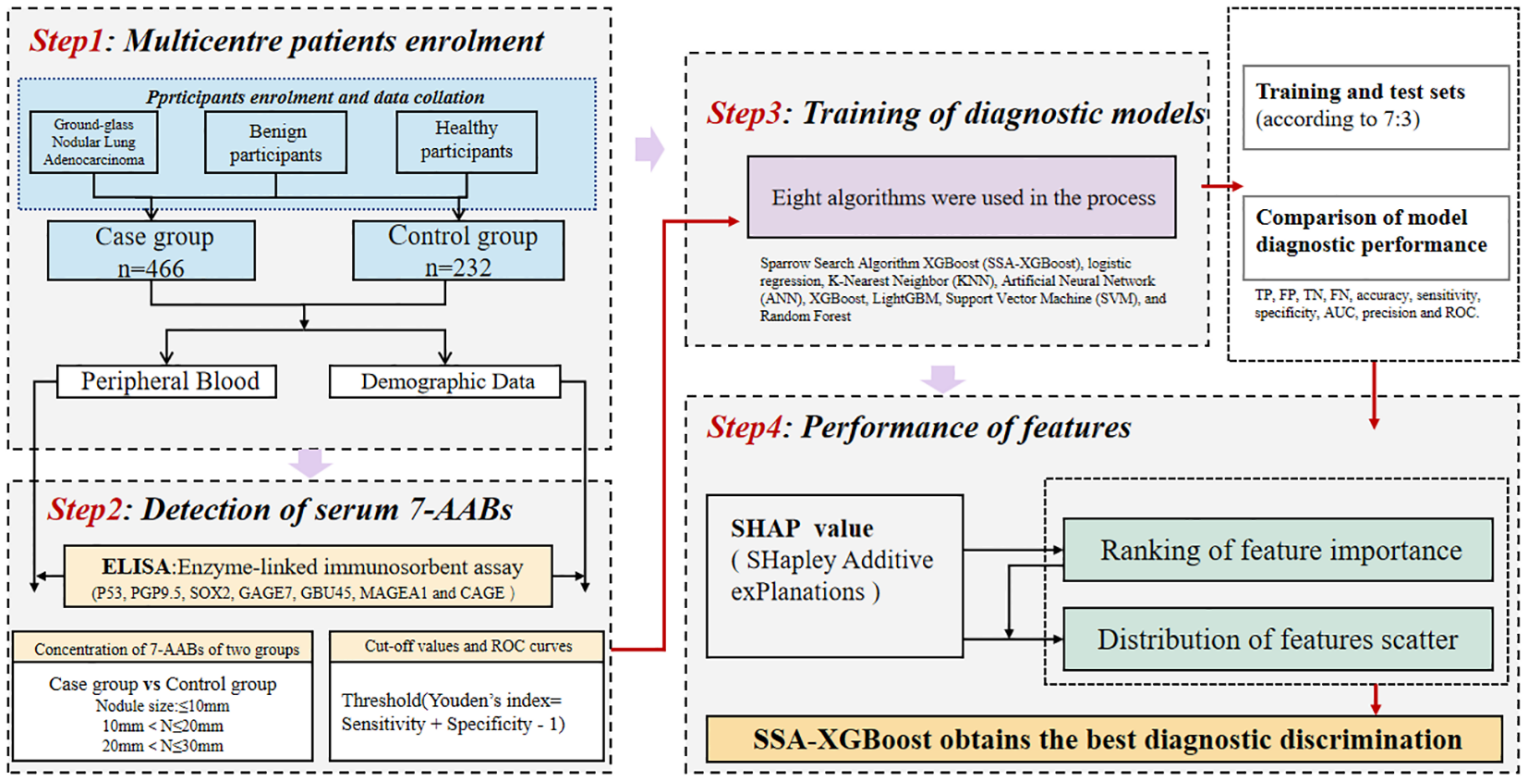
Flowchart of inclusion and grouping in this study.

In the process of constructing the machine learning model, participants were divided into two sets: a training set and a testing set (allocated in a 7:3 ratio). The training set data are utilized for model fitting, while the test set is employed to evaluate the accuracy of the trained model (Training set: n=488, test set: n=210). Eight algorithms were employed in this process: Sparrow Search Algorithm-XGBoost (SSA-XGBoost), logistic regression, K-Nearest Neighbor (KNN), Artificial Neural Network (ANN), XGBoost, LightGBM, Support Vector Machine (SVM), and Random Forest. To assess the diagnostic performance of the models, the following metrics were utilized: sensitivity, specificity, precision, classification accuracy, and area under the curve (AUC), among others. Furthermore, the SHapley Additive explanation (SHAP) values were utilized to evaluate the extent to which each feature contributes to the diagnostic performance of early-stage ground-glass nodular lung adenocarcinoma. Finally, we used the Hosmer-Lemeshow test to assess the calibration of the binary classification model and performed decision curve analysis for all models. The study design process is illustrated in **Figure 2**.

## Results

### Study population

A total of 698 participants were enrolled in this prospective clinical trial, with 466 individuals in the case group, comprising 175 males and 291 females, the majority of whom were female (62.4%) and non-smokers (79.0%). The case group consisted of patients with early-stage ground-glass nodular lung adenocarcinoma, diagnosed by histopathological methods, with 60.3% of the patients having mGGNs. The pathological types were categorized as minimally invasive adenocarcinoma (MIA), invasive adenocarcinoma (IAC), and precursor lesions based on histopathological patterns, with IAC accounting for 69.9%. The remaining subjects comprised healthy participants or patients with benign GGNs. The disease types diagnosed in the benign nodule group included pulmonary infections, benign pulmonary nodules, interstitial pneumonia, and pulmonary fungal infections. The clinical characteristics of the subjects are summarized in **Table 1**.

**Table 1:** Basic information of study population: abc in the table indicates that there is a difference between groups.

	Case group (n=466)	Benign group (n=90)	Healthy group (n=142)	P value
Gender, n (%)				
Male	175 (37.6)^a^	49 (54.4)	71 (50)^c^	<0.05
Female	291 (62.4)	41 (45.6)	71 (50)	
Age (median, range)	57 ± 11^a^	58 ± 15^b^	39 ± 14^c^	<0.05
Smoking history, NO (n,%)	368 (79.0)	66 (73.3)	112 (78.8)	>0.05
Pathological type				
Precursor lesion	27 (5.8)	0 (0)	0 (0)	
MIA	113 (24.2)	0 (0)	0 (0)	
IAC	326 (69.9)	0 (0)	0 (0)	
Size of nodule, n (%)				
A (N ≤ 1cm)	192 (41.2)	42 (46.6)	0 (0)	
B (1cm<N ≤ 2cm)	218 (46.7)	19 (21.1)	0 (0)	
C (2cm<N ≤ 3cm)	56 (12.0)	13 (14.1)	0 (0)	
D (N>3cm)	0 (0)	16 (17.7)	0 (0)	
Diseases, n (%)				
Lung Adenocarcinoma	466 (100)	0 (0)	0 (0)	
pulmonary infection	0 (0)	41 (45.6)	0 (0)	
Benign pulmonary nodule	0 (0)	45 (50.0)	0 (0)	
Interstitial pneumonia	0 (0)	3 (3.3)	0 (0)	
Pulmonary fungal infection	0 (0)	1 (1.1)	0 (0)	
Healthy population	0 (0)	0 (0)	142 (100)	

a, there is a difference between the case group and the benign group; b, there is a difference between the benign group and the healthy group; c, there is a difference between the case group and the healthy group.

### The serum concentration of seven autoantibodies

**Figure 3:**
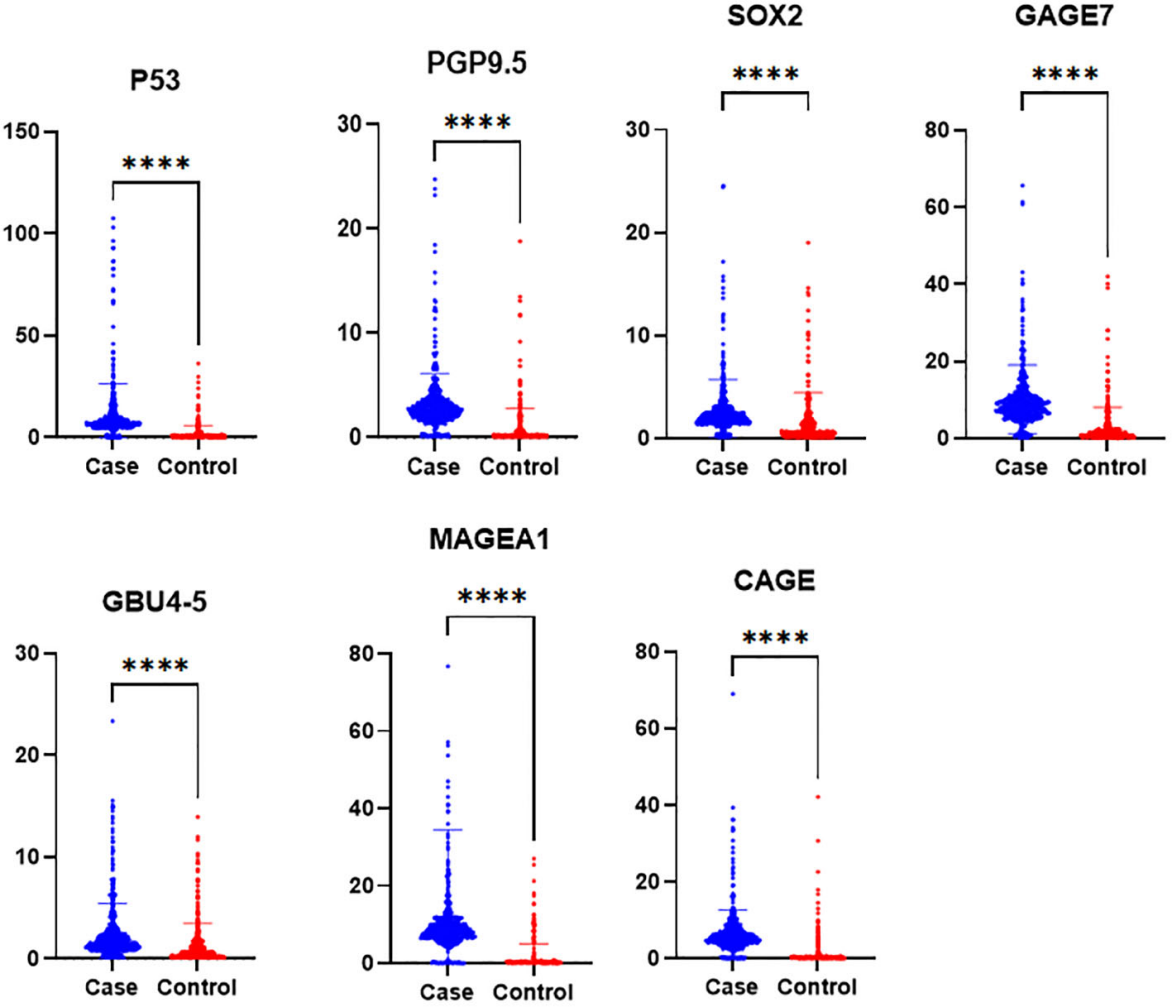
The serum concentration of seven autoantibodies in the case and control groups. P-values less than 0.0001 are marked with [****].

Serum samples from the study population were analyzed for the levels of seven antibodies, including P53, PGP9.5, SOX2, GAGE7, GBU45, MAGEA1, and CAGE. The patients were initially divided into three groups: the case group, the benign nodule group, and the healthy group. Differences in the expression levels of the seven autoantibodies among the three groups were compared, followed by a pairwise comparison among the groups (**Table 2**). Subsequently, the benign nodule group and the healthy population were categorized as a control group, and differences between the control and case groups were compared. Levels of the seven antibodies were significantly higher in the case group than in the control group (**Table 3**; **Figure 3**). Further analysis indicated that, within the case group, there was no significant difference in the expression levels of the seven antibodies between patients in the mGGNs and pGGNs groups; however, the mean levels in the mGGNs group were higher than those in the pGGNs group.

**Table 2:** The serum concentration of seven autoantibodies among the three groups. The superscript abc in the table indicates that there is a difference between groups.

	Case group (n=466)	Benign group (n=90)	Healthy group (n=142)	P value
P53, u/ml M (P25, P75)	7.14 (5.77,10.33)^a^	0.96 (0.35,6.28)	0.70 (0.30,1.20)^c^	<0.05
PGP9.5, u/ml M (P25, P75)	2.68 (2.03,3.51)^a^	0.50 (0.14,2.03)	0.20 (0.10,0.50)^c^	<0.05
SOX2, u/ml M (P25, P75)	2.05 (1.57,2.79)^a^	0.80 (0.40,1.95)	1.00 (0.40,1.80)^c^	<0.05
GAGE7, u/ml M (P25, P75)	8.30 (6.19,10.94)^a^	1.95 (0.77,7.65)^b^	1.30 (0.70,2.20)^c^	<0.05
GBU4_5, u/ml M (P25, P75)	1.61 (1.06,2.65)	1.18 (0.30,2.50)^b^	0.70 (0.40,1.30)^c^	<0.05
MAGEA1, u/ml M (P25, P75)	8.56 (6.66,10.96)^a^	0.23 (0.10,8.11)	0.20 (0.10,0.30)^c^	<0.05
CAGE, u/ml M (P25, P75)	5.37 (4.10,7.29)^a^	0.11 (0.10,4.76)^b^	0.20 (0.10,0.40)^c^	<0.05

a, there is a difference between the case group and the benign group; b, there is a difference between the benign group and the healthy group; c, there is a difference between the case group and the healthy group.

**Table 3:** The serum concentration of seven autoantibodies among two groups.

	Case group (n=466)	Control group (n=232)	P
P53, u/ml M (P25, P75)	7.14 (5.77,10.33)	0.80 (0.33,1.79)	<0.0001
PGP9.5, u/ml M (P25, P75)	2.68 (2.03,3.51)	0.20 (0.10,0.80)	<0.0001
SOX2, u/ml M (P25, P75)	2.05 (1.57,2.79)	0.90 (0.40,1.85)	<0.0001
GAGE7, u/ml M (P25, P75)	8.30 (6.19,10.94)	1.50 (0.71,2.88)	<0.0001
GBU4_5, u/ml M (P25, P75)	1.61 (1.06,2.65)	0.80 (0.30,1.70)	<0.0001
MAGEA1, u/ml M (P25, P75)	8.56 (6.66,10.96)	0.20 (0.10,0.50)	<0.0001
CAGE, u/ml M (P25, P75)	5.37 (4.10,7.29)	0.20 (0.10,0.60)	<0.0001

We also categorized ground-glass nodular lung adenocarcinoma into three groups: Group A (nodule size 1 cm or less in greatest dimension), Group B (nodule size more than 1 cm but no more than 2 cm in greatest dimension), and Group C (nodule size more than 2 cm but no more than 3 cm in greatest dimension), in accordance with tumor size, and assessed the expression of each of the seven autoantibodies. There were no significant differences in autoantibody expression among the three patient groups. However, the four antibodies, P53, GBU4_5, MAGEA1, and CAGE, demonstrated a trend of increased expression as the nodule diameter increased.

### Determination of the cut-off values of seven autoantibodies

**Figure 4:**
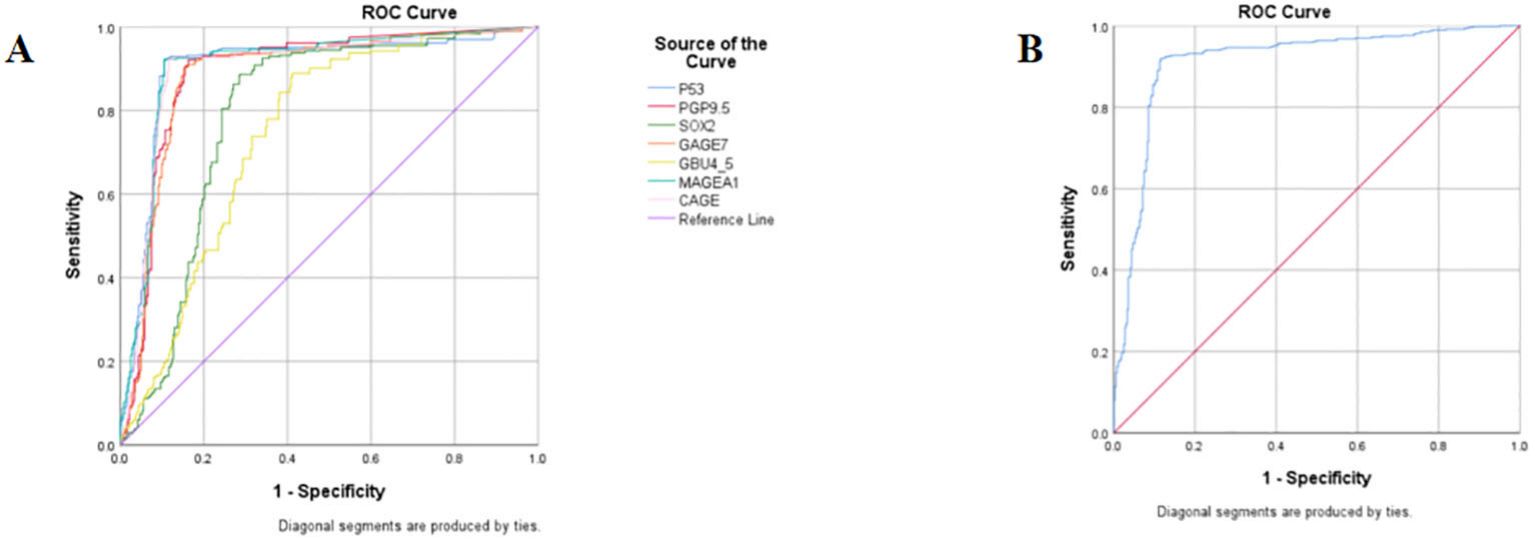
ROC curves of seven autoantibodies. **(A)** ROC curves for 7 autoantibodies used individually; **(B)** ROC curves for 7 antibodies are combined for diagnosis.

Diagnostic experiments utilizing receiver operating characteristic (ROC) curves enabled the determination of cut-off values for seven autoantibodies. Each group of antibodies demonstrated good sensitivity and specificity for the diagnosis of ground-glass nodular lung adenocarcinoma, with P53, MAGEA1, PGP9.5, and CAGE achieving an area under the curve (AUC) of approximately 0.87. Furthermore, ROC analyses indicated that the combined diagnostic approach was more effective than the individual tests, achieving a sensitivity of 84.05% and a specificity of 91.85% (**Table 4**; **Figure 4**).

**Table 4:** The cut-off values of seven autoantibodies, sensitivity and specificity of each antibody when applied to differential diagnosis individually.

	Cut off	Sensitivity	Specificity	AUC
P53	3.509	84.05	92.27	0.8705
PGP9.5	1.219	78.45	92.06	0.8629
SOX2	1.401	64.66	86.27	0.7489
GAGE7	4.083	80.17	90.99	0.8563
GBU4_5	0.9002	56.03	84.33	0.7130
MAGEA1	2.921	85.34	92.27	0.8763
CAGE	1.997	83.62	92.27	0.8709
Combined seven antibodies		84.05	91.85	0.8870

### Seven antibodies and model diagnosis

**Figure 5:**
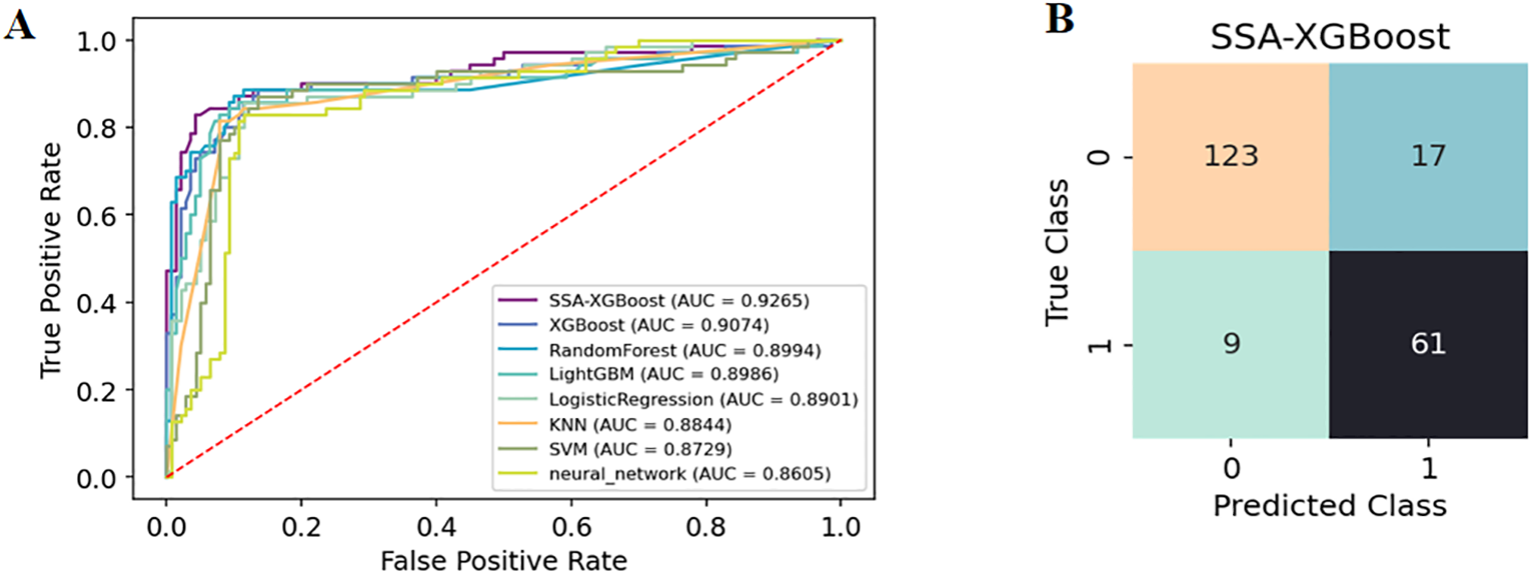
**(A)** The diagnostic ROC curves for each of the eight machine learning models. **(B)** SSA-XGBoost’s Confusion Matrix.

**Figure 6:**
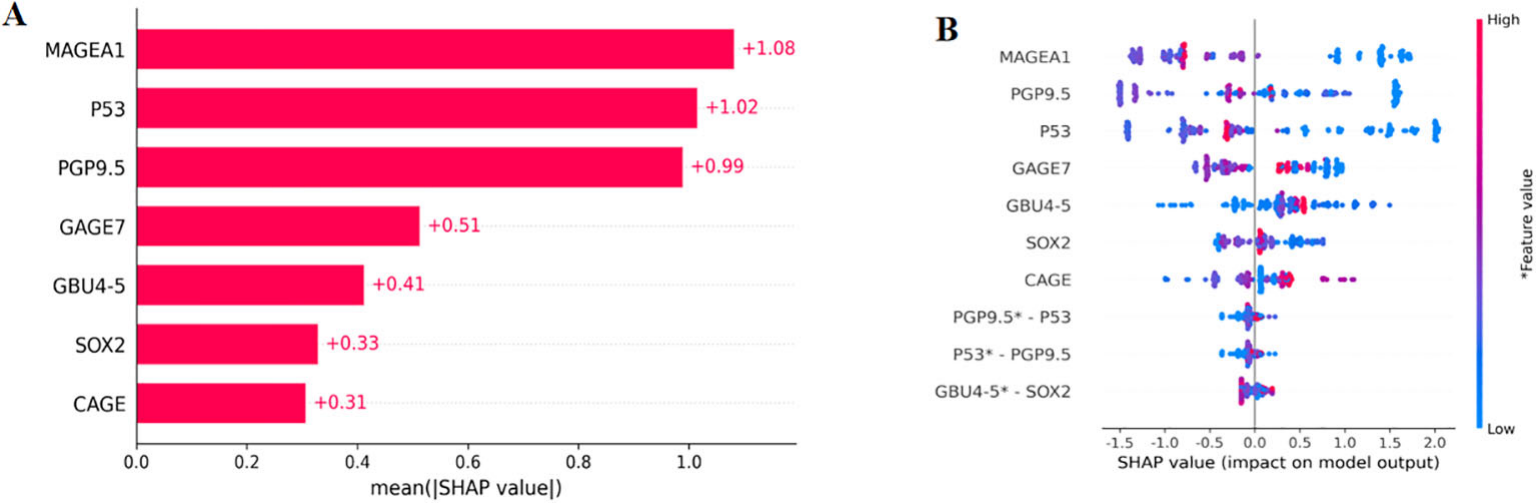
SHAP, SHapley Additive explanation. SHAP summary plot for seven autoantibodies contributing to the SSA-XGBoost model. **(A)** Ranking of feature importance indicated by SHAP. The matrix plot depicts the importance of each covariate in the development of the final predictive model. **(B)** The attributes of the features in the black box model. Each line represents a feature, and the abscissa is the SHAP value. Red dots represent higher feature values, and blue dots represent lower feature values.

The enrolled population was divided into a training set (n=488) and a test set (n=210) in a 7:3 ratio. Eight models were constructed in this study, and their predictive effects were compared: SSA-XGBoost, XGBoost, LightGBM, K-Nearest Neighbors (KNN), Logistic Regression, Random Forest (RF), Artificial Neural Network (ANN), and Support Vector Machine (SVM). The comparison included true positives (TP), false positives (FP), true negatives (TN), false negatives (FN), accuracy, sensitivity, specificity, area under the curve (AUC), and precision, and ROC curves were plotted for each model (**Table 5**; **Figure 5**). During model construction, XGBoost achieved the highest AUC values in the training set, while SSA-XGBoost achieved the highest AUC values in the testing set. The best-performing model was the Sparrow Search Algorithm-XGBoost model, which achieved an AUC of 0.9265, with sensitivity and specificity of 0.8714 and 0.8786, respectively, in the test set (**Figure 5**). SHAP analysis revealed that the most contributing features among the seven autoantibodies were MAGEA1, P53, and PGP9.5 (**Figure 6**).

**Table 5:** Machine learning models used for ground-glass nodular lung adenocarcinoma early detection based on the metabolomic biomarker features.

	TP	FP	TN	FN	Accuracy	Sensitivity	Specificity	AUC	Precision
Training set									
SSA-XGBoost	155	5	321	7	0.9754	0.9568	0.9847	0.9982	0.9688
XGBoost	162	1	325	0	0.9980	1.0000	0.9969	1.0000	0.9939
LightGBM	138	20	306	24	0.9098	0.8519	0.9387	0.9780	0.8734
KNN	133	20	306	29	0.8996	0.8210	0.9387	0.9560	0.8693
Logistic Regression	138	24	302	24	0.9016	0.8519	0.9264	0.8888	0.8519
RF	140	22	304	21	0.9098	0.8642	0.9325	0.9435	0.8642
ANN	122	26	300	40	0.8648	0.7531	0.9202	0.8707	0.8243
SVM	139	22	304	23	0.9078	0.8580	0.9325	0.9128	0.8634
Test set									
SSA-XGBoost	61	17	123	9	0.8762	0.8714	0.8786	0.9265	0.7821
XGBoost	60	18	122	10	0.8667	0.8571	0.8714	0.9074	0.7692
LightGBM	60	16	124	10	0.8762	0.8571	0.8857	0.8986	0.7895
KNN	57	13	127	13	0.8711	0.8814	0.8623	0.9097	0.8455
Logistic Regression	59	16	124	11	0.8714	0.8429	0.8857	0.8901	0.7867
RF	62	16	124	8	0.8857	0.8857	0.8857	0.8994	0.7949
ANN	57	15	125	13	0.8667	0.8143	0.8929	0.8605	0.7919
SVM	58	16	124	12	0.8667	0.8286	0.8857	0.8729	0.7838

SSA-XGBoost, Sparrow Search Algorithm XGBoost; KNN, K-NearestNeighbor; RF, Random Forests; ANN, Artificial Neural Network; SVM, support vector machines; TP, true positive; FP, false positive; TN, true negative; FN, false negative; AUC, area under the curve.

**Figure 7:**
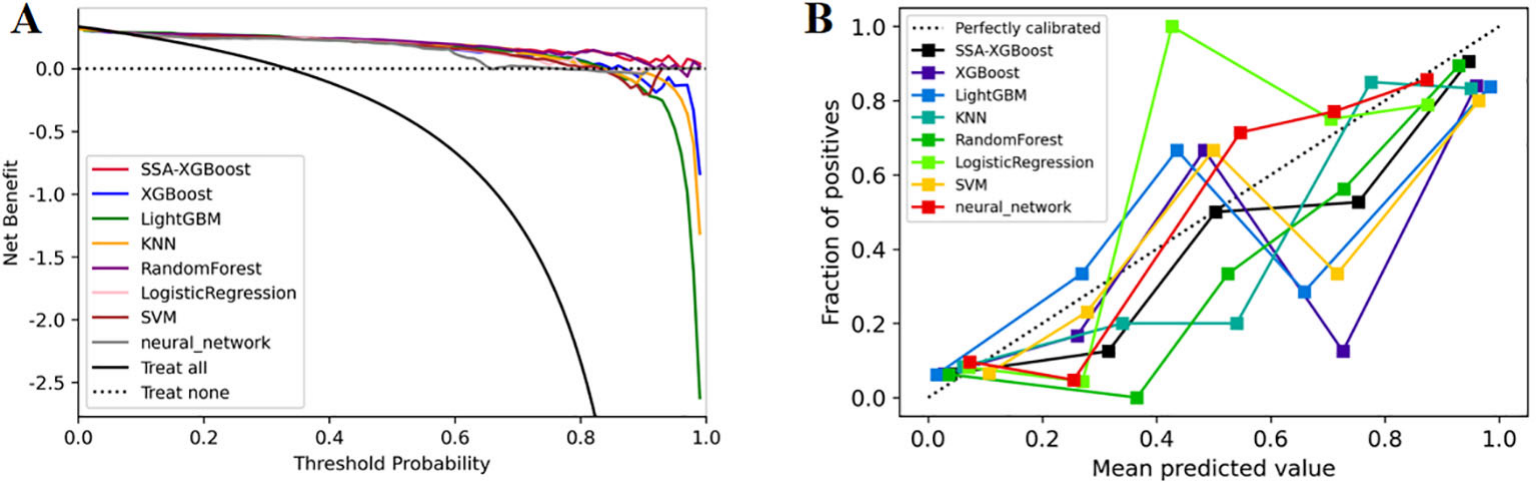
Model evaluation: **(A)** Decision curve analysis; **(B)** Calibration curves.

In addition, we evaluated the models using decision curve analysis and assessed the calibration of all machine learning models in the study (**Figure 7**). The calibration plot demonstrated the consistency between the predicted probabilities of lung adenocarcinoma for ground-glass nodules and the actual probabilities. According to the decision curve analysis, the net benefit area of the SSA-XGBOOST model was the largest. In summary, our findings indicate that the SSA-XGBOOST model has clinical utility.

## Discussion

In this prospective study, we measured the serum levels of seven autoantibodies in participants from several medical centers in China. The seven autoantibodies demonstrated excellent specificity and sensitivity in identifying ground-glass nodular lung adenocarcinoma early(≤3cm), achieving 91.58% specificity and 84.05% sensitivity when combined for this diagnosis. Furthermore, an AUC of 0.9265 and accuracy was 0.8762 were obtained in our constructed machine learning diagnostic model. In addition, MAGEA1, P53, and PGP9.5 were confirmed as the three most informative features in the model using SHAP analysis.

Pulmonary ground-glass nodules on CT imaging are typically classified as pure ground-glass nodules (pGGNs) and mixed ground-glass nodules (mGGNs) (7). Studies have indicated that symptomatic ground-glass nodules (37% of pGGNs and 48% of mGGNs) regress or disappear within three months, suggesting that these may be inflammatory nodules (7, 18). Early-stage lung cancer presenting with ground-glass nodules differs from traditional lung cancer with solid nodules in terms of biological behavior, including the degree of malignancy, the rate of progression, prognosis, and even causative factors. Smoking is strongly associated with lung cancer; however, in real-world studies, it is not a direct risk factor in the population with ground-glass nodule lung adenocarcinoma, as confirmed by several studies (1, 7). In our study population, which included patients with ground-glass nodular lung adenocarcinoma and benign nodules, there was a higher proportion of non-smokers. Furthermore, the diagnosis and follow-up of ground-glass nodules are controversial, with different treatments recommended for various sizes of ground-glass nodules according to the Fleischner Guidelines and European Guidelines (19–21). The NCCN recommends a follow-up period of 12 months for mixed ground-glass nodules less than 6 mm, while the Fleischner Guidelines state that nodules smaller than 6 mm do not require follow-up (19, 22). In actual clinical practice, however, the majority of ground-glass nodules remain unchanged for extended periods. The protracted and gradual progression of ground-glass nodules complicates effective management, necessitating further exploration of non-invasive markers. Therefore, we aim to provide reference evidence regarding the controversial aspects of non-invasive markers. Previous imaging studies of pulmonary nodules have suggested that the likelihood of malignant transformation is significantly higher in nodules exceeding 3 cm in diameter (23). Consequently, nodules larger than 3 cm have been the primary focus of numerous studies. In our study, the patients in the enrolled case group presented with nodules smaller than 3 cm. We investigated the critical values of autoantibodies in patients with nodules of this size, thereby providing reference values for early diagnosis. It has been suggested that mixed ground-glass nodules are more aggressive than pure ground-glass nodules; therefore, we compared the autoantibody levels between the pGGNs and mGGNs groups to observe the differing immune responses of these two types of nodules (24). However, we found that the mean levels of antibody expression were higher in the mGGNs group than in the pGGNs group; nonetheless, there was no statistically significant difference. This may be attributed to the absence of a correlation between antibody levels and tumor aggressiveness. Furthermore, the ground-glass nodules selected in our study were at an early stage, suggesting that the immune response may not yet be sufficient to effectively address their aggressiveness.

Multiple proteins of tumor cells induce auto-reactive immune responses in cancer patients due to various modifications such as glycosylation, phosphorylation, oxidation and protein hydrolysis and cleavage, and the antibodies that are really produced against these proteins are cancer autoantibodies (25, 26), marking their importance as molecular signatures of useful clinical diagnostic and prognostic information. Most of the autoantibodies found in the sera of cancer patients target cellular proteins associated with modifications, aberrant localization or expression related to cell cycle progression, signal transduction, proliferation and apoptosis (25).

Additionally, the expression of these autoantibodies has been confirmed in other studies to be associated with tumor progression and prognosis. GBU4_5 can control the methylation process of transposons during cell differentiation and suppress gene expression (17). The abnormal expression of CAGE autoantibodies is significantly negatively correlated with the overall survival of lung cancer patients (27). In our study, we also observed that the expression of partial autoantibodies increased with the size of the nodules, which is consistent with previous research findings. However, our experimental data did not indicate significant differences, which may be related to the characteristics of our study population. It has been demonstrated that autoantibody combinations for diagnosis are significantly more sensitive and specific than traditional biomarkers (including CEA, NSE and CYFRA21-1) (13). And in the overall lung cancer population, seven autoantibodies (p53, PGP9.5, SOX2, GAGE7, GBU4-5, MAGEA1, CAGE) also obtained good sensitivity and specificity (28). Similarly, in our study population, the seven autoantibodies differed significantly between the case and control groups with good diagnostic performance.

The rise in the detection of lung nodules has resulted in an increase in the analysis of various types of medical data, thereby placing a greater burden on the medical profession. The integration of machine learning and deep learning into the clinical application of lung cancer diagnosis has the potential to enhance the efficiency of both diagnosis and treatment. Furthermore, it serves as a tool for the multimodal integration of imaging information and laboratory data. A computer-aided diagnosis (CAD) system utilizing random forest (RF) algorithms has been developed to identify benign and malignant nodules, achieving sensitivities of 92.4% and specificities of 94.8%, respectively (29). The model PLCOm2012 (30), designed to facilitate lung cancer screening using routine laboratory data and demographic information, demonstrated an area under the curve (AUC) of 0.80 for the operating characteristics of subjects in both the development and validation datasets. The MES model developed by Michael K. Gould et al. demonstrated a 53.0% improvement in sensitivity while maintaining specificity (31). The presence of lung cancer-associated autoantibodies significantly enhances diagnostic accuracy when utilized in conjunction with CT or other laboratory data (28, 32). In our study, machine learning models developed for ground-glass nodules smaller than 3 cm—including patients with early-stage ground-glass nodular lung adenocarcinoma, benign nodules, and healthy individuals—yielded clear diagnostic results when combined with SSA-XGBOOST. This advancement enhances the diagnostic capabilities for nodules of this size. The performance of the machine learning models regarding feature importance is somewhat unexplainable; therefore, we report SHAP values to present a ranked list of the impact of the seven autoantibodies. SHAP values are primarily used to quantify the contribution of each feature to model predictions, thereby helping to elucidate the “black box” nature of various models (33). This implies that physicians should prioritize the expression levels of the more clinically relevant antibodies.

In summary, the experimental data from patients with ground-glass nodular lung adenocarcinoma used for model training were sourced from multiple medical centers, thereby enhancing the model’s generalizability to some extent. Our study established a new set of cut-off values for ground-glass nodular lung adenocarcinoma and validated them within the developed machine learning model. This represents the first investigation of autoantibodies against lung adenocarcinoma in patients with ground-glass nodules smaller than 3 cm. We aim to integrate additional biomarkers to minimize the risk of over-medication in lung cancer screening, which shows promise for distinguishing between benign and malignant lung nodules. We anticipate that more validated biomarkers will emerge to facilitate the early diagnosis of lung cancer.

## Data Availability

The raw data supporting the conclusions of this article will be made available by the authors, without undue reservation.
